# Drugs involved in Kentucky drug poisoning deaths and relation with antecedent controlled substance prescription dispensing

**DOI:** 10.1186/s13011-023-00561-y

**Published:** 2023-09-01

**Authors:** Patricia R. Freeman, Jana McAninch, Nabarun Dasgupta, Douglas R. Oyler, Krassimir Slavov, Candice Collins, Sarah Hargrove, Edward Freeman, Dustin Miracle, Svetla Slavova

**Affiliations:** 1 Department of Pharmacy Practice and Science, College of Pharmacy, University of Kentucky, Lexington, KY USA; 2 Office of Surveillance and Epidemiology, Center for Drug Evaluation and Research, U.S. Food and Drug Administration, Silver Spring, MD USA; 3 Injury Prevention Research Center, Gillings School of Global Public Health, University of North Carolina, Chapel Hill, NC USA; 4 Department of Biostatistics, College of Public Health, University of Kentucky, Lexington, KY USA; 5 Kentucky Injury Prevention and Research Center, University of Kentucky, Lexington, KY USA; 6 Department of Epidemiology, College of Public Health, University of Kentucky, Lexington, KY USA

**Keywords:** Drug poisoning, Overdose death, Opioid, Prescription monitoring program, Stimulant

## Abstract

**Background:**

The shift from prescription to illicit drugs involved in drug poisoning deaths raises questions about the current utility of prescription drug monitoring program (PDMP) data to inform drug poisoning (overdose) prevention efforts. In this study, we describe relations between specific drugs involved in Kentucky drug poisoning deaths and antecedent controlled substance (CS) dispensing.

**Methods:**

The study used linked death certificates and PDMP data for 2,248 Kentucky resident drug poisoning deaths in 2021. Death certificate literal text analysis identified drugs mentioned with involvement (DMI) in drug poisoning deaths. We characterized the concordance between each DMI and the CS dispensing history for this drug at varying timepoints since 2008.

**Results:**

Overall, 25.5% of all decedents had dispensed CS in the month before fatal drug poisoning. Over 80% of decedents were dispensed opioid(s) since 2008; the percentage was similar regardless of opioid involvement in the poisoning death. One-third of decedents had dispensed buprenorphine for treatment of opioid use disorder since 2008, but only 6.1% had dispensed buprenorphine in the month preceding death. Fentanyl/fentanyl analogs were DMI in 1,568 (69.8%) deaths, yet only 3% had received a fentanyl prescription since 2008. The highest concordance in the month preceding death was observed for clonazepam (43.6%).

**Conclusion:**

Overall, concordance between CS dispensing history and the drugs involved in poisoning deaths was low, suggesting a need to reevaluate the complex relationships between prescription medication exposure and overdose death and to expand harm reduction interventions both within and outside the healthcare system to reduce drug poisoning mortality.

**Supplementary Information:**

The online version contains supplementary material available at 10.1186/s13011-023-00561-y.

## Introduction

A decade ago, seminal research using prescription drug monitoring program (PDMP) data and other administrative dispensing records was instrumental in establishing associations between opioid prescribing patterns and drug poisoning (often called “drug overdose”) mortality. [1–5] Now, the changing landscape of drug involvement in drug poisoning deaths – from prescription opioids to illicit fentanyl - raises important questions about the utility of PDMP data to identify and intervene with individuals at risk for drug poisoning. However, as PDMPs have been operational for at least a decade in most states, the accumulated data provide a rich source of information on both recent and remote exposure to prescription controlled substances (CS) that can inform research on potential causal pathways between exposure and drug poisoning [6, 7]. Differentiating these causal pathways is critical to policy and intervention design, but the practical inability to link prescribing of specific substances with drug poisoning toxicology hinders such analyses [8].

The development of programming tools to analyze death certificate literal text [9, 10] allows the identification of specific drugs mentioned with involvement (DMI) in drug poisoning death and holds considerable promise to generate more directly actionable insights. The analysis of specific DMI and relationship with antecedent CS dispensing requires a linkage between drug poisoning death certificate and PDMP records. While currently not possible at the national level, state or local-level studies have analyzed linked cross-sectional datasets, providing an opportunity to examine different root causes of drug poisoning [11–18]. The use of static cross-sectional linked datasets, however, precludes ongoing surveillance. As most drug poisonings are now due to illicitly manufactured opioids, outdated policies that do not address this shift may exacerbate drug poisoning risk from unregulated street drugs [19].

In 2018, the Kentucky Cabinet for Health and Family Services built an automated process within Kentucky’s PDMP (Kentucky All Schedule Prescription Electronic Reporting [KASPER]), connecting all newly filed death certificates with the decedent’s CS prescription history. Quarterly surveillance reports track changes in drug poisoning mortality by demographic, intent, involved drugs/drug classes, and drug-specific concordance with 90-day prescription history using literal text analysis [20]. This surveillance system revealed a significant jump in drug poisoning deaths in the second quarter of 2020 (coincident with COVID-19 pandemic disruptions), followed by sustained high mortality through the end of 2021, leading to a call to action for harm reduction outreach and naloxone access to those using stimulants [21, 22].

In the present study, we relate specific DMI to antecedent CS dispensing among Kentucky drug poisoning decedents. We focus on concordance between DMI and dispensing history 1, 3, and 6 months prior to death, and ever (i.e., any dispensing recorded in KASPER since its inception in 2008), as well as time between last prescription and death.

## Methods

### Data sources and data linkage

The study used linked records from two data sources:

1. Electronic death certificate records from the Kentucky Office of Vital Statistics (extracted in April 2022). The records contained all fields from the US Standard Certificate of Death [23], including the literal text describing cause-of-death, significant contributing conditions and how the injury occurred, as well as assigned ICD-10 (International Classification of Diseases and Related Health Problems, Tenth Revision) codes for the underlying cause-of-death (UCD) and up to 20 multiple (i.e., contributing) causes-of-death [24–26].
2. KASPER records from the Cabinet of Health and Family Services on CS prescriptions (Schedules II-V) dispensed at Kentucky retail pharmacies, or via internet or mail order pharmacies to a Kentucky residence address.

### Study sample and measures

The study sample consisted of 2,248 Kentucky resident drug poisoning decedents (hereafter, decedents) who died in 2021 of drug poisoning, captured as death certificate records with UCD codes X40-X44 (unintentional drug poisoning), X60-X64 (suicide), X85 (homicide), or Y10-Y14 (undetermined intent) [27] Drug poisoning deaths were classified as opioid-involved (based on presence of ICD-10 multiple cause-of-death codes in the range T40.0-T40.4 or T40.6) and/or stimulant-involved (ICD-10 codes T40.5 for cocaine, or T43.6 for other psychostimulants) [27].

### Drugs mentioned with involvement in drug poisoning deaths

Specific DMI in drug poisoning deaths were identified by analyzing the literal text in the death certificate records using National Center for Health Statistics and U.S. Food and Drug Administration (FDA) DMI methodology [10, 28, 29]. Drug search terms were cross-walked to *principal variants* and further cross-walked to *referent drug groups. A referent drug group* includes all principal variants representing the drug, its metabolites, precursors, and analogues. A version of the DMI software program is available on GitHub [30]. For manuscript nomenclature, we retain the capitalization schema used in the original software to differentiate the constructed referent drug group (FENTANYL), from verbatim text (“fentanyl”), and the substance name (fentanyl).

### CS dispensing history

Using MEDI-SPAN generic product identifier (GPI) codes, [31] a 14-digit number that allows identification of drug products by primary and secondary classifications, we identified drug classes and substances of interest for this study based on historical patterns of use, diversion, and drug poisoning involvement (see Additional File 1):

1. Drug classes/groups: benzodiazepines; buprenorphine products approved by the FDA as medications for treatment of opioid use disorder (MOUD; excluding buprenorphine for pain); gabapentinoids; opioid analgesics (not including opioid antitussives); centrally acting muscle relaxants; pharmaceutical stimulants and z-drugs.
2. Products containing the following substances: alprazolam, amphetamine/dextroamphetamine, buprenorphine, clonazepam, diazepam, fentanyl, gabapentin, hydrocodone, methadone, methamphetamine, morphine, oxycodone, and tramadol. It should be noted that methadone dispensed at opioid treatment programs was not reported to KASPER during the timeframe of this study (2008–2021), thus methadone prescriptions identified in dispensing histories were prescriptions dispensed for the treatment of pain.
  We constructed indicators using four time points based on the date of the last dispensed CS: 1, 3, and 6 months prior to death, and ever (i.e., between the earliest date for KASPER data collection, January 1, 2008, and the date of death). The monthly intervals were determined with the SAS^®^ function INTNX. Additionally, we calculated the time since the last dispensing, by specific class and substance, defined as the number of days between death and the last dispensed prescription within the corresponding drug class or substance.

## Analysis

Data were summarized with frequencies and percentages. Pearson χ2 test was used for association between categorical variables. Counts ≤ 5 and rates based on ≤ 10 events were suppressed per the state data reporting policy. Mortality rates per 100,000 residents used 2021 population estimates [32].

We characterized drugs involved in drug poisoning deaths and concordance with decedents’ CS dispensing history, as well as days between the last dispensing of an involved prescription drug and death.

### Ethics statement

The University of Kentucky Institutional Review Board approved the study (Protocol # 47384) and granted a waiver of the informed consent process as the study uses retrospectively obtained secondary data of decedents, only aggregated numbers are reported, with frequencies ≤ 5 and rates based on ≤ 10 events suppressed according to state vital statistics data reporting policy.

## Results

### Demographic factors and history of prescription opioid use

The study sample included 2,248 Kentucky resident drug poisoning deaths in 2021 (provisional data), a rate of 49.9/100,000 (Table 1). Most decedents were male (67.6%) and Non-Hispanic White (87.9%). Almost one third of the decedents were aged 35–44, with an age-specific death rate of 119/100,000. Those identified as Non-Hispanic Black experienced the highest drug poisoning death rate among all racial/ethnic groups at 61.2/100,000, 17% higher compared to Non-Hispanic White (52.5/100,000).

Opioids were involved in 1,787 (79.5%) drug poisoning deaths. Almost half of the opioid-involved drug poisoning deaths also involved cocaine or other stimulants (n = 888) (Table 1). Residents aged 35–44 years had the highest fatality rate for opioid without stimulant (43.3/100,000) and opioid plus stimulant (54.3/100,000). Male (27.8/100,000) and Non-Hispanic Black residents (30.0/100,000) had the highest opioid plus stimulant-involved drug poisoning death rates.

Overall, 1,874 (83.4%) decedents had at least one dispensed opioid prescription recorded since KASPER inception (Table 1). This percentage was similar for opioid-involved and non-opioid-involved deaths. 85% of Non-Hispanic White decedents received an opioid prescription, compared to 74.0% of Non-Hispanic Black decedents and 24.1% of Hispanic decedents (Table 1).

The majority (96.4%) of drug poisoning deaths were unintentional, 2.1% were intentional/suicide (there were no homicide drug poisoning deaths), and 1.5% were classified as undetermined intent. We compared the history of dispensed prescriptions in the month before death as well as since 2008 for the three groups of drug poisoning deaths (unintentional, suicide, and undetermined intent) for any opioids, benzodiazepines, stimulants, gabapentinoids, muscle relaxants, z-drugs, and buprenorphine MOUD. The only differences we found were (1) in the history of dispensed buprenorphine MOUD since 2008 (34% for unintentional poisoning decedents, 20.6% undetermined, 16.7% suicide; p = 0.01), and (2) the last month dispensing of benzodiazepines (20.8% suicide, 8.8% undetermined, and 8.4% unintentional; p = 0.01).

**Table 1:** Characteristics of Kentucky residents who died from drug poisoning in 2021, involvement of opioids and stimulants in the drug poisoning deaths, and history of any dispensed opioid prescriptions

Drug Poisoning Death Characteristics	Drug Poisoning Deaths					Dispensed^b^ opioid prescriptions after January 1, 2008 N(row %)
	N(col %)	Rate (per 100,000 residents) by involved drug				
		Any drug	Opioid^a^ without stimulant (n = 899)	Opioid and stimulant (n = 888)	Stimulant without opioid (n = 258)	
All	2248	49.9	19.9	19.7	5.7	1874(83.4)
Age at death						
<18	12(0.5)	1.2	supp^c^	supp	supp	supp
18–24	130(5.8)	31.4	17.6	11.1	supp	supp
25–34	485(21.6)	82.3	34.6	35.8	7.0	374(77.1)
35–44	670(29.8)	119.0	43.3	54.3	10.7	568(84.8)
45–54	489(21.8)	87.7	32.1	34.3	13.4	436(89.2)
55–64	372(16.5)	62.1	26.2	18.7	10.7	326(87.6)
65+	90(4.0)	11.7	4.7	2.7	supp	80(88.9)
Race/ethnicity						
White NH	1977(87.9)	52.5	21.0	20.2	6.2	1689(85.4)
Black NH	231(10.3)	61.2	23.1	30.0	6.4	171(74.0)
Other NH	11(0.5)	6.2	supp	supp	supp	7(63.6)
Hispanic	29(1.3)	15.4	8.0	5.9	supp	7(24.1)
Sex						
Male	1510(67.2)	67.6	25.9	27.8	8.1	1229(81.4)
Female	738(32.8)	32.4	14.1	11.8	3.4	645(87.4)
Drug poisoning Intent^d^						
Unintentional	2166(96.4)	48.0	19.2	19.4	5.5	1812(83.7)
Intentional	48(2.1)	1.1	0.3	supp	supp	37(77.1)
Undetermined	34(1.5)	0.8	0.4	supp	supp	25(73.5)

^a^Opioid involved drug poisoning death is defined as a drug poisoning death (i.e., underlying cause-of-death in the ICD-10 range X40-X44, X60-X64, X85, Y10-Y14) and a multiple cause-of-death in the ICD-10 range T40.0-T40.4 or T40.6. Stimulant-involved drug poisoning death is defined as a drug poisoning death with involvement of cocaine or other psychostimulants (e.g., amphetamine, methamphetamine), and captured as a death certificate with an underlying cause-of-death in the ICD-10 range X40-X44, X60-X64, X85, Y10-Y14 and a multiple cause-of-death ICD-10 code T40.5 or T43.6

^b^Definition for dispensed opioid analgesics products (MEDI-SPAN search criteria) is provided in Appendix Table A1

^c^Counts ≤ 5 and rates based on ≤ 10 events are suppressed by state data reporting policy. The next smallest count is suppressed to prevent calculation of the suppressed small count by subtraction from the total

^d^Drug poisoning intent is determined based on the underlying cause-of-death ICD-10 codes: Unintentional (X40-X44), Intentional (X60-X64, X85), Undetermined Intent (Y10-Y14)

### Prescription drugs involved in drug poisoning deaths, and history of involved drug dispensing

The top 13 prescription DMIs are listed in Table 2. The referent drug group FENTANYL includes fentanyl, fentanyl analogs and 4-ANPP (precursor for illicitly manufactured fentanyl). FENTANYL was mentioned with involvement in 1,568 (69.8%) deaths. Only 3% (n = 47) of the decedents had any reported dispensed prescriptions for fentanyl.

**Table 2:** History of controlled substance prescription dispensing for Kentucky residents who died from drug poisoning in 2021 (n = 2248), by specific drug mentioned with involvement on the death certificate

Drug mentioned with involvement in drug poisoning deaths^a^	N(%^d^)	Dispensing history^e^ for the specific drugs mentioned with involvement, by time interval preceding the death			
		1 month prior	3 months prior	6 months prior	Since 2008
		N(%^f^)	N(%^f^)	N(%^f^)	N(%^f^)
FENTANYL^b^	1,568(69.8)	≤ 5^ g^	≤ 5	≤ 5	47(3.0)
METHAMPHETAMINE	937(41.7)	0	0	0	0
AMPHETAMINE	391(17.4)	13(3.3)	16(4.1)	16(4.1)	36(9.2)
GABAPENTIN^c^	259(11.5)	104(40.1)	114(44.0)	125(48.3)	163(62.9)
OXYCODONE	146(6.5)	54(37.0)	60(41.1)	61(41.8)	108(74.0)
ALPRAZOLAM	133(5.9)	36(27.1)	36(27.1)	37(27.8)	68(51.1)
HYDROCODONE	123(5.5)	41(33.3)	49(39.8)	59(48.0)	111(90.2)
MORPHINE	97(4.3)	0	0	0	10(10.3)
CLONAZEPAM	78(3.5)	34(43.6)	36(46.2)	37(47.4)	50(64.1)
DIAZEPAM	59(2.6)	19(32.2)	20(33.9)	21(35.6)	35(59.3)
TRAMADOL	54(2.4)	11(20.4)	15(27.8)	16(29.6)	31(57.4)
BUPRENORPHINE	49(2.2)	19(38.8)	22(44.9)	22(44.9)	33(67.3)
METHADONE^h^	40(1.8)	≤ 5	≤ 5	≤ 5	≤ 5

^a^Deaths involving HEROIN (n = 58; 2.6%) or COCAINE (n = 220; 9.8%) were not included in Table 2 as they do not have corresponding prescription drug products that would be dispensed and reported to the Kentucky All Schedule Prescription Electronic Reporting (KASPER) system

^b^The referent group FENTANYL included fentanyl and fentanyl analogs mentioned with involvement in drug poisoning deaths. The reported prescription history for decedents with fentanyl/fentanyl analog- involved deaths is for prescription fentanyl (see Appendix, Table A1, for MEDI-SPAN Search Criteria). The interpretation of the data is that for example, 47 (3%) of the decedents who experienced fentanyl/fentanyl analog- involved drug poisoning deaths had a prescription for fentanyl dispensed after January 1, 2008

^c^Gabapentin became a Schedule V controlled substance in Kentucky, reportable to KASPER on July 1, 2017

^d^Percent of the total number of drug poisoning deaths (n = 2248)

^e^Definitions for selecting the drug products (MEDI-SPAN search criteria) are provided in Table A1 (see Additional File 1)

^f^Row percent

^g^Counts greater than 0 but ≤ 5 were suppressed according to the state data reporting policy; Percentages are not reported in the case of a suppressed cell count

^h^Methadone for treatment of opioid use disorder is not reported to KASPER

Note: The drug involved groups are not mutually exclusive; a death that involved multiple substances is reported under each relevant reference drug

The other two most mentioned opioids were OXYCODONE (n = 146; 6.5%) and HYDROCODONE (n = 123; 5.5%), with concordance in prescription dispensing during the month preceding death of 37% and 33.3%, respectively. More than 90% of decedents with HYDROCODONE-involved drug poisoning deaths had prescriptions for hydrocodone dispensed since 2008. While deaths involving METHADONE were rare (n = 40; 1.8%), ≤ 5 of these decedents had a methadone prescription recorded in KASPER at any point (methadone dispensed in MOUD treatment programs was not reported to KASPER during the study period). Morphine was not dispensed in the 6 months before death for the decedents with MORPHINE-involved drug poisoning deaths.

METHAMPHETAMINE was the second most frequently listed DMI (41.7% of drug poisoning deaths) but no decedents had a history of being dispensed prescription methamphetamine; however, 9.2% of the decedents with AMPHETAMINE-involved deaths had a history of prescription amphetamine receipt.

The highest concordance in the month preceding death (Table 2) was observed for CLONAZEPAM (43.6%), GABAPENTIN (40.1%), and BUPRENORPHINE (38.8%). In the six months preceding death, about one-half of decedents with GABAPENTIN (48.3%) or HYDROCODONE (48.0%) involved deaths received prescriptions for the involved pharmaceutical product.

We did not report concordance with prescription history for deaths with HYDROMORPHONE (0.8%) or OXYMORPHONE (1.6%) DMI because they were not among the leading drugs mentioned with involvement and because these substances are also metabolites of hydrocodone and oxycodone, respectively. In our study, 94.4% of OXYMORPHONE DMI also listed OXYCODONE DMI; 77.8% of the deaths with HYDROMORPHONE DMI also listed HYDROCODONE DMI.

### General history of CS dispensing in drug poisoning decedents

Overall, 25.5% of all drug poisoning decedents had some dispensed CS in the month before the death, which increased to 40% and 87% when extending the lookback period to 6 months and to January 1, 2008, respectively (Table 3). The most dispensed drug class was opioid analgesics, with 83.4% of decedents ever having a dispensed prescription and 11.6% having a prescription dispensed in the month preceding death. The second most frequently dispensed class was benzodiazepines, with 46.6% of decedents receiving a prescription after January 1, 2008. Approximately one-third of drug poisoning decedents (33.1%, n = 744) had history of dispensed buprenorphine MOUD prescriptions, but only 6.1% (n = 138) had a dispensed prescription within the month preceding death. Almost 11% (n = 243) of decedents had a gabapentinoid prescription in the month prior to death; of these, almost all (91.8%) were for gabapentin (data not shown). There was little difference in the prescription history by drug class of those who died from unintentional vs. intentional drug poisoning (within 3%) except for the benzodiazepine class, where dispensed prescriptions were noted in 8% of unintentional drug poisoning deaths vs. 20.8% of intentional drug poisoning deaths (data not shown).

**Table 3:** General history of dispensed controlled substance prescriptions by drug class for all Kentucky drug poisoning decedents who died in 2021 (n = 2248)

Dispensed prescription, by class^a^	History of dispensed controlled substance prescriptions in relation to the day of the drug poisoning death			
	1 month prior	3 months prior	6 months prior	Any time after January 1, 2008
	N(%)	N(%)	N(%)	N(%)
**Benzodiazepines**	186(8.3)	220(9.8)	247(11.0)	1048(46.6)
**Buprenorphine MOUD**	138(6.1)	207(9.2)	284(12.6)	744(33.1)
**Gabapentinoid** ^**b**^	243(10.8)	304(13.5)	354(15.7)	728(32.4)
**Muscle Relaxant**	< 5	< 5	< 5	139(6.2)
**Opioid Analgesics** ^**c**^	260(11.6)	364(16.2)	459(20.4)	1874(83.4)
**Stimulant**	35(1.6)	43(1.9)	48(2.1)	191(8.5)
**Z-drugs**	22(1.0)	32(1.4)	35(1.6)	316(14.1)
**Any controlled substance**	574(25.5)	752(33.5)	900(40.0)	1956(87.0)

^a^Definitions for selecting the drug products (MEDI-SPAN search criteria) is provided in Additional File 1

^b^Prior to 2017, the gabapentinoid class contained only pregabalin; gabapentin became a Schedule V controlled substance in Kentucky, reportable to KASPER on July 1, 2017

^c^Excluding buprenorphine-containing products

**Fig. 1:**
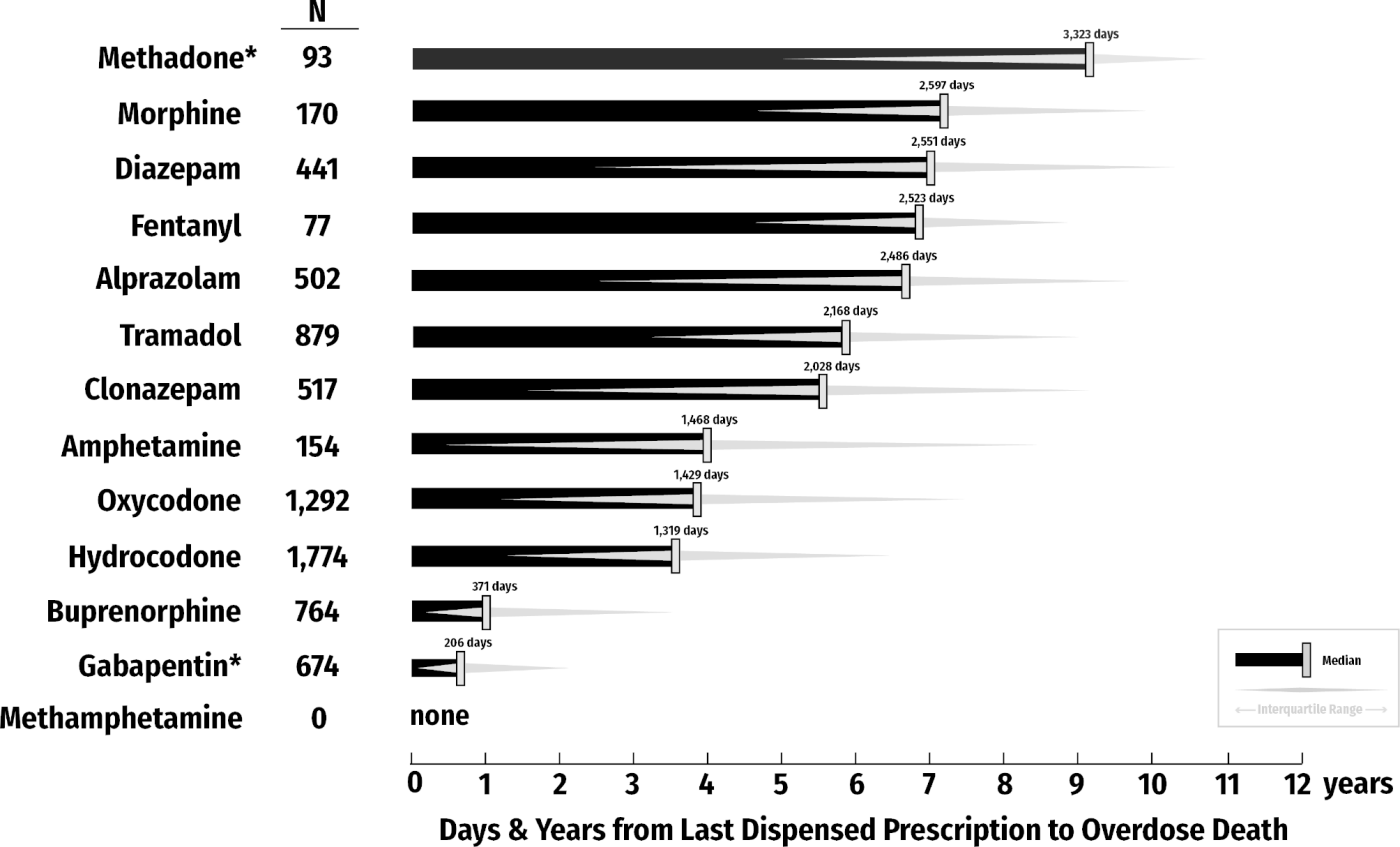
Median time (years/days from the last dispensed prescription for specific medications to drug poisoning deaths, Kentucky drug poisoning decedents, 2021 * Methadone for treatment of opioid use disorder is not reported to the Kentucky All Schedule Prescription Electronic Reporting (KASPER) system. Gabapentin became a Schedule V controlled substance in Kentucky, reportable to KASPER on July 1, 2017 Note: The reported number of decedents with history of specific dispensed controlled substance includes everyone with such history, regardless of the involvement of the substance in the drug poisoning death

The elapsed time from last dispensed CS to drug poisoning death is presented in Fig. 1. Of the 2,248 drug poisoning decedents, 1,774 (78.9%) had a history of dispensed hydrocodone, and 1,292 (57.5%) of oxycodone, but the median time since the last dispensing was around 4 years. Decedents with dispensed buprenorphine prescription (n = 764; 33.9%) had more recent history, with median time 371 days (IQR: 78 to 1293) since the last dispensed prescription. Prescription dispensing for other medications was remote, with median time of almost a decade for dispensed methadone (n = 93; 4.1%).

### CS dispensing, by type of opioid involved in the drug poisoning death

There were 223 drug poisoning deaths that involved prescription opioids, without involvement of FENTANYL or HEROIN (Table 4). In the 6 months preceding death, 63.2% of these decedents had dispensed CSs, nearly one-half (45.3%) had dispensed opioid analgesics, and 22.9% received benzodiazepines. A smaller percentage of decedents with FENTANYL- or HEROIN-involved deaths had dispensed opioid analgesic (16.5%) or benzodiazepine (8.6%) prescription in the 6 months prior to death. For decedents with drug poisoning deaths not involving opioids, 21.7% received an opioid analgesic prescription in the 6 months preceding death and 13.5% received a benzodiazepine.

**Table 4:** Drug poisoning decedents with dispensed prescriptions for selected drug classes, by drugs involved in the drug poisoning deaths, and in relation to the day of the drug poisoning death (N = 2248)

Controlled substances, by class^a^	Deaths involving FENTANYL or HEROIN (N = 1573)			Deaths involving opioids other than FENTANYL or HEROIN (N = 223)				Deaths not involving opioids^b^ (N = 452)		
	1 month prior	6 months prior	Since 2008	1 month prior	6 months prior	Since 2008	1 month prior		6 months prior	Since 2008
	N(%)	N(%)	N(%)	N(%)	N(%)	N(%)	N(%)		N(%)	N(%)
Buprenorphine MOUD	101(6.4)	222(14.1)	585(37.2)	16(7.2)	24(10.8)	53(23.8)	21(4.6)		38(8.4)	106(23.5)
Opioid Analgesics	123(7.8)	260(16.5)	1299(82.6)	83(37.2)	101(45.3)	196(87.9)	54(11.9)		98(21.7)	379(83.8)
Benzodiazepine	99(6.3)	135(8.6)	678(43.1)	43(19.3)	51(22.9)	140(62.8)	44(9.7)		61(13.5)	230(50.9)
Stimulants	25(1.6)	36(2.3)	141(9.0)	≤ 5	≤ 5	12(5.4)	8(1.8)		10(2.2)	38(8.4)
Muscle Relaxants	≤ 5	≤ 5	78(5.0)	0	0	31(13.9)	0		≤ 5	30(6.6)
Z-drugs	12(0.8)	18(1.1)	184(11.7)	≤ 5	7(3.1)	55(24.7)	6(1.3)		10(2.2)	77(17.0)
Gabapentinoids	123(7.8)	188(12.0)	449(28.5)	65(29.1)	82(36.8)	123(55.2)	55(12.2)		84(18.6)	156(34.5)
Any controlled subst.	340(6.0)	582(37.0)	1361(86.5)	120(53.8)	141(63.2)	203(91.0)	114(25.2)		177(39.2)	392(86.7)

^a^Definitions for selecting the drug products (MEDI-SPAN search criteria) are provided in Additional File 1

^b^These deaths involved only non-opioid drug(s) (e.g., methamphetamine n = 219, cocaine n = 31, gabapentin n = 30, alprazolam n = 11; not mutually exclusive: a death that involved both methamphetamine and gabapentin is counted under each drug)

Note: The number of drug poisoning deaths with FENTANYL involvement was based on literal text analysis. There were 9 drug poisoning deaths involving FENTANYL that were not coded with an ICD-10 code for synthetic opioid and were not classified as opioid-involved deaths in Table 1

Note: Counts greater than 0 but ≤ 5 were suppressed according to the state data reporting policy; Percentages are not reported in the case of a suppressed cell count

## Discussion

Dispensing-data linked with electronic death certificates allowed us to relate specific drug involvement in drug poisoning deaths to antecedent CS prescription dispensing among Kentucky residents who died in 2021. Similar to the national pattern, drug poisoning deaths in Kentucky continue to be driven by opioids, specifically fentanyl and its analogs, with almost half of opioid-involved poisoning deaths also involving cocaine or other stimulants. Non-Hispanic Blacks had the highest rates of stimulant involvement, as has been reported previously [33] Exposure to synthetic fentanyl and analogues through use of contaminated illicit stimulant drugs presents a new complexity when considering short and long-term causal pathways to fatal drug poisoning and highlights the importance of expanding naloxone access to those using stimulants. While relatively uncommon from 2011 to 2016, [34] recent data from 77 harm reduction programs in 25 states that submitted samples to a mail-in drug checking service at the University of North Caroline showed that the overall adjusted prevalence of fentanyl in stimulant samples was 12% for powder methamphetamine and 15% for powder cocaine [35].

Similar to findings about opioid-detected poisoning deaths investigated in Connecticut 2016–2017, [36] most decedents did not have recently dispensed prescriptions for the CS listed as DMI, indicating either use of diverted prescription CSs or exposure to illicitly manufactured drugs. Decedents with HYDROCODONE-involved poisoning deaths had the highest percentage of being dispensed the involved drug at some point since January 2008 (90.2%), which is not surprising given the ubiquity of hydrocodone usage among adults; of the 62.7 mil opioid doses dispensed in Kentucky in the second quarter of 2022 (most recent data currently available), 27.9 mil (44.4%) were hydrocodone doses [37]. Less than 5 decedents with FENTANYL-involved poisoning deaths had dispensed fentanyl prescriptions in the 6 months preceding the deaths. For comparison, 16% of the 2013–2014 Kentucky drug poisoning decedents with positive toxicology for fentanyl had a fentanyl prescription in the 30 days before the overdose deaths [17]. No decedent with MORPHINE-involved poisoning death had recent prescriptions for morphine, suggesting that the morphine listed on the death certificate is either diverted, or more likely, a metabolite of heroin detected postmortem. Following use, heroin rapidly crosses the blood brain barrier and is quickly metabolized to 6-monoacetylmorphine and then to morphine [38]. For comparison, among the 2013–2014 Kentucky drug poisoning decedents with morphine present at the time of the death, 4% had a recently dispensed prescription for morphine [39]. It should be noted that drug poisoning deaths with HEROIN DMI in Kentucky continue to decline, representing only 5.6% of deaths in 2020, [22] and 2.6% deaths (58 out of 2248) in 2021.

Overall, one in four Kentucky drug poisoning decedents had dispensed CS prescriptions in the month prior to the death indicating contact with the health care system and perhaps a missed opportunity for intervention. This is similar to what has previously been reported for 2017 decedents in Vermont (30%) [40] but lower than the older results reported for 2016 decedents in West Virginia (40%; 332 out of 830) [41]. Alternatively, three in four Kentucky drug poisoning decedents did not have this indicator of recent health system contact, highlighting the need to support and expand interventions outside the health care system (e.g., community-based harm reduction, drug courts) to reach persons at high risk of fatal drug poisoning.

While one third (n = 744) of all decedents historically filled prescriptions for buprenorphine MOUD, only 6.1% (n = 138) had a dispensed buprenorphine MOUD prescription in the month prior to death. This dramatic difference shows the importance of treatment retention and calls for further investigation into the circumstances for discontinuation to highlight barriers for OUD treatment in Kentucky. For example, there was a significant drop in the number of Kentucky residents in buprenorphine treatment for OUD during the first few weeks after the COVID-19 public health emergency declaration, [42] coinciding with a jump in opioid poisoning mortality [22]. Very few decedents with METHADONE-involved poisoning had received methadone from a pharmacy at any point since 2008, suggesting that the methadone was either from an opioid treatment program or diverted, but rarely from their own prescription for treatment of pain.

There are multiple potential causal pathways between drug exposure and drug poisoning death. Early in therapy, toxicity could arise from patient behavior, such as accidentally or intentionally taking more than prescribed [43, 44]. Alternatively, short-term toxicity could arise from prescriber behavior, such as high starting doses [45] or unsafe initiation of high potency opioids in opioid-naïve patients [45–47]. One longer-term causal pathway hypothesizes that iatrogenic exposure predisposes patients to opioid use disorder, and patients eventually overdose from leftover, diverted, or illicitly manufactured opioids [48, 49]. Yet another pathway suggests drug poisonings occur when patients’ long-term opioid therapy is abruptly tapered or stopped, which may occur as a result of supply limiting polices, predisposing individuals to accidental or intentional drug poisoning from nonmedical opioid use [50–55]. A fifth potential pathway involves patients with pre-existing unmanaged substance use disorders who are exposed to prescription opioids iatrogenically with limited clinical guardrails, accelerating drug poisoning risk [56–59].

While we did not set out to test causal pathways as formal statistical hypotheses, examining the time elapsed between the last dispensed prescription and the day of the death provides directions for investigations in the future. For example, the median times between the last prescription and drug poisoning deaths for two drugs, HYDROCODONE and OXYCODONE, were 1,319 and 1,429 days, respectively. Only about one-third of decedents with HYDROCODONE or OXYCODONE toxicity had a prescription for that substance in the month prior to death, and these deaths accounted for only 7% and 6% of drug poisoning mortality, respectively (not mutually exclusive groups). Further, MORPHINE- and FENTANYL-involved poisoning deaths were rarely associated with dispensed prescriptions for that substance. However, 83.4% of the decedents had a history of dispensed opioid analgesics. Collectively, when iatrogenic prescription drug exposure mechanisms leading to drug poisoning death are assessed, our data support complex, varied, and often protracted causal pathways rather than immediate toxicity as the main drivers of drug poisoning mortality in patients with dispensed opioids. These findings are not incompatible with some causal pathways to drug poisoning involving iatrogenic opioid analgesic exposure, given that over 80% of decedents had received prescription(s) for an opioid analgesic since 2008; however, the broadly similar opioid dispensing histories for drug poisoning deaths involving opioids (particularly fentanyl and/or heroin) and those involving only non-opioids belie simplistic causal inferences about opioid exposure and drug poisoning risk and highlight the complexity of the current drug poisoning crisis.

Our analysis is subject to limitations. For example, decedent prescriptions filled in other states were not captured. Additionally, the drugs mentioned as involved in drug poisoning deaths were extracted from the literal text of the death certificates as recorded by the coroners certifying the death. It is possible that prescription medications were present in post-mortem toxicology results but were not listed as DMI by coroners. Due to the complex nature of drug metabolism and overlap between parent drugs and metabolites, it is possible that some DMI recorded by corners were actually metabolites. For example, morphine is a known metabolite of heroin, and amphetamine is a metabolite of methamphetamine; thus, MORPHINE DMI might indicate heroin involvement, and AMPHETAMINE DMI- methamphetamine involvement. In these cases, no referent drug prescription would be expected.

## Conclusion

Drugs involved in drug poisoning deaths generally did not align with drugs dispensed in the weeks and months before death; however, remote prescription opioid dispensing was common among decedents from both opioid and non-opioid drug poisonings. These findings suggest that for many individuals, PDMP data may have limited utility for drug poisoning prevention. Furthermore, the near universal history of remote exposure to opioids challenges us to reevaluate the causal pathways between prescription medication exposure and drug poisoning death. Interaction with the health care system provides opportunities for screening, intervention, and treatment, but expansion of harm reduction interventions outside the healthcare system is critical to reach most of those at risk of fatal drug poisoning.

### Electronic supplementary material

Below is the link to the electronic supplementary material.

Supplementary Material 1

## Data Availability

The data used in this study is not available for posting in a public repository due to the conditions of data use agreements with the Kentucky All Schedule Prescription Electronic Program and the Kentucky Office of Vital Statistics.
