# Supplementary material for: Drugs involved in Kentucky drug poisoning deaths and relation with antecedent controlled substance prescription dispensing
Source: Subst Abuse Treat Prev Policy. 2023 Sep 1;18:53. doi: 10.1186/s13011-023-00561-y (PMC10474700; doi:10.1186/s13011-023-00561-y)
Supplement: Supplementary file 1 — Supplementary Material 1 [file 13011_2023_561_MOESM1_ESM.docx]

**Drugs Involved in Kentucky Poisoning Deaths and Relation with Antecedent Controlled Substance Prescription Dispensing**

**Additional File 1**: **Drug Product Selection Criteria**

***Table A1: Definitions for Selecting Drug Products for Characterizing Drug Overdose Decedents’ History of Dispensed Controlled Substances***

|  | Definition (SQL MEDI-SPAN Search Criteria^‡^) |
| --- | --- |
| **Class** |  |
| Amphetamines | TCGPI_ID like ‘6110%’ |
| Benzodiazepines | TCGPI_ID like ‘7210%’ or ‘5710%’ or ‘602010%’ |
| Central Muscle Relaxants | TCGPI_ID like ‘7510%’ |
| Gabapentinoids | TCGPI_ID like ‘7260003000%’ or ‘6256003020%’ or ‘7260005700%’ |
| Opioid Analgesics^§^ | TCGPI_ID like ‘6510%’ or ‘6599%’ or ‘6520%’ & TCGPI_NAME not like ‘buprenorphine’ |
| Z Hypnotics | TCGPI_ID like ‘602040%’ |
| Buprenorphine  MOUD | TCGPI_ID like ‘6520001%’ & EFFICACY_CODE not containing ‘U’ & ROUTE_OF_ADMINISTRATION not like ‘IJ’ OR ‘TD’ & TCGPI_NAME not like ‘Buprenorphine HCL Buccal Film’ or ‘Probuphine’ |
| **Drugs** |  |
| Alprazolam | TCGPI_NAME like ‘alprazolam’ |
| Amphetamine/ Dextroamphetamine | TCGPI_NAME like ‘amphetamine’ and not like ‘methamphetamine’ OR ‘hydroxyamphetamine’ |
| Buprenorphine | TCGPI_NAME like ‘buprenorphine’ |
| Clonazepam | TCGPI_NAME like ‘clonazepam’ |
| Diazepam | TCGPI_NAME like ‘diazepam’ |
| Fentanyl | TCGPI_NAME like ‘fentanyl’ |
| Gabapentin | TCGPI_NAME like ‘gabapentin’ |
| Hydrocodone | TCGPI_NAME like ‘hydrocodone’ |
| Methadone | TCGPI_NAME like ‘methadone’ |
| Methamphetamine | TCGPI_NAME like ‘methamphetamine’ |
| Morphine | TCGPI_NAME like ‘morphine’ and not like ‘apomorphine’ |
| Oxycodone | TCGPI_NAME like ‘oxycodone’ |
| Tramadol | TCGPI_NAME like ‘tramadol’ |
| MOUD = Medication for Opioid Use Disorder  ^§^Excluding buprenorphine-containing products  ^‡^TCGPI_ID corresponds to generic product identifier (GPI) codes, linking individual drug products to their categorical class; TCGPI_NAME corresponds to the drug name and is inclusive of all drug products containing that drug ingredient; EFFICACY_CODE refers to the drug product’s FDA determination, with U referring to ‘undeterminable’; ROUTE_OF_ADMINISTRATION refers to the drug product’s approved route of administration, with IJ referring to ‘injectable’ and TD referring to ‘transdermal’  % denotes that search term can contain any character afterwards | |

Wolters Kluwer. Medi-Span Generic Product Identifier (GPI). Accessed June 30, 2022, <https://www.wolterskluwer.com/en/solutions/medi-span/about/gpi>
